# Temperature-Driven Local Acclimatization of *Symbiodnium* Hosted by the Coral *Galaxea fascicularis* at Hainan Island, China

**DOI:** 10.3389/fmicb.2017.02487

**Published:** 2017-12-12

**Authors:** Guowei Zhou, Lin Cai, Yuanchao Li, Haoya Tong, Lei Jiang, Yuyang Zhang, Xinming Lei, Minglan Guo, Sheng Liu, Pei-Yuan Qian, Hui Huang

**Affiliations:** ^1^Key Laboratory of Tropical Marine Bio-resources and Ecology, Guangdong Provincial Key Laboratory of Applied Marine Biology, South China Sea Institute of Oceanology, Chinese Academy of Sciences, Guangzhou, China; ^2^Tropical Marine Biological Research Station in Hainan, Sanya Joint Laboratory of Marine Science Research, Chinese Academy of Sciences, Sanya, China; ^3^Shenzhen Research Institute and Division of Life Science, Hong Kong University of Science and Technology, Hong Kong, Hong Kong; ^4^Hainan Academy of Ocean and Fisheries Sciences, Haikou, China

**Keywords:** coral, *Symbiodinium*, symbiosis, diversity, flexibility

## Abstract

The success of coral reef ecosystems largely depends on mutualistic symbiosis between scleractinian corals and the dinoflagellate photosymbiont *Symbiodinium* spp. However, further investigation is needed to elucidate the flexibility of coral-algae associations in response to environmental changes. In this study, we applied a molecular method (high-throughput internal transcribed spacer 2 region of ribosomal RNA gene amplicon sequencing) to explore diversity and flexibility of *Symbiodinium* associated with *Galaxea fascicularis*, an ecologically important scleractinian coral species collected at five locations around Hainan Island, South China Sea. The results revealed a high diversity of *Symbiodinium* subclades with C2r and D17 being dominant in *G. fascicularis*. Clade D *Symbiodinium* occurred most frequently in habitats where the annual average sea surface temperatures are the highest, suggesting that temperature is an important factor in determining *Symbiodinium* D abundance in *G. fascicularis*. The distribution of coral-*Symbiodinium* associations are possibly mediated by trade-off mechanisms which change the relative abundance of *Symbiodinium* clades/subclades under different environmental conditions. These findings provide further evidence that reef-building corals such as *G. fascicularis* can shuffle their symbionts to cope with environmental changes, and have implications for our understanding of the ecology of flexible coral-algal symbiosis.

## Introduction

The success of coral reef ecosystems in oligotrophic ocean depends largely on mutualistic symbioses between reef-building corals and photosymbiontic algae of the genus *Symbiodinium* (zooxanthellae). *Symbiodinium* is comprised of nine phylogenetic clades (A–I), each containing multiple genetically distinct subclades or species (Baker, 2003; Pochon and Gates, 2010). Reef-building corals readily form associations with clades A–D *Symbiodinium*, but partnerships with clades F and G have also been reported (Lajeunesse, 2001; Baker, 2003; Lajeunesse et al., 2010a). The functional and physiological diversity within *Symbiodinium* (Tchernov et al., 2004; Brading et al., 2011) strongly affects the response of coral holobionts to environmental disturbances. Heat tolerant *Symbiodinium* in clade D, for example, may strengthen the thermal tolerance of corals exposed to heat stress (Berkelmans and Van Oppen, 2006; Lajeunesse et al., 2009; Keshavmurthy et al., 2012). Therefore, reef-building corals that harbor multiple ecologically distinct *Symbiodinium* clades or types are expected to be flexible and have more opportunities to cope with environmental change (Baker, 2003; Little et al., 2004; Berkelmans and Van Oppen, 2006) or mediate their sensitivity to stress (Putnam et al., 2012).

Coral reefs are in serious decline worldwide as a result of global warming and anthropogenic activities (Pandolfi et al., 2011). However, it has been hypothesized that corals could adapt to environmental perturbations by either shuffling existing symbionts or switching to novel symbionts (Buddemeier and Fautin, 1993; Baker, 2003; Baker et al., 2004). Knowing whether corals can associate flexibly with a range of symbionts is a necessary prerequisite to test this hypothesis. Changes in the symbiont communities associated with scleractinan corals have been observed following disturbance and are presumed to be an important mechanism for acclimatization (Jones et al., 2008; Silverstein et al., 2015). For instance, an increase in *Symbiodinium* D1a has been previously reported following bleaching events in Pacific and Caribbean corals (Baker et al., 2004; Lajeunesse et al., 2009, 2010b). Moreover, flexible symbiosis in conspecific and congeneric corals has been shown to be related to both depth and geographical distribution (Sampayo et al., 2007; Lajeunesse et al., 2010a; Huang et al., 2011; Lien et al., 2013).

Progress in surveying *Symbiodinium* diversity and ecology has considerably improved our understanding of the flexibility of coral-algae symbiosis (e.g., Baker, 2003; Baird et al., 2007; Silverstein et al., 2012). However, most previous studies only considered the dominant *Symbiodinium* types because of the limitations of conventional screening approaches (e.g., Lajeunesse et al., 2010a; Zhou and Huang, 2011). More recently, as high-resolution methods including quantitative PCR and next-generation DNA sequencing have been increasingly used (e.g., Mieog et al., 2009; Arif et al., 2014; Boulotte et al., 2016; Ziegler et al., 2017), evidence for some corals hosting unusual or rare *Symbiodinium* is increasing. These less common symbionts have the potential to influence the whole holobiont function, including bleaching resilience (Mieog et al., 2009; Silverstein et al., 2012; Arif et al., 2014; Thomas et al., 2014; Cunning et al., 2015c; Boulotte et al., 2016). Therefore, coral-algal symbioses may be more flexible than previously thought and need to be investigated urgently to provide a better understanding of how flexibility in coral holobionts enables them to cope with environmental changes.

The coral species *Galaxea fascicularis* (Linnaeus, 1767) is broadly distributed in the Indo-Pacific region and is an ecologically important species in the South China Sea. Each generation of *G. fascicularis* acquires symbiotic algae horizontally and harbors multiple *Symbiodinium* clades or types, commonly clades C and/or D (Lajeunesse et al., 2010a; Huang et al., 2011). Previous studies have shown that *Symbiodinium* associated with *G. fascicularis* is flexible with respect to both clades C and D at regional (Huang et al., 2011) and local scales (Zhou et al., 2012) in the South China Sea. However, coral-algal associations are also dependent on local physicochemical conditions (Lajeunesse et al., 2010a; Howells et al., 2012). Hainan Island is the largest island in the South China Sea and the coral reefs are affected by environmental conditions, such as coast and summer upwelling (Jing et al., 2015). In the present study, we investigated the *Symbiodinium* communities associated with *G. fascicularis* around Hainan Island using internal transcribed spacer 2 (ITS2) region of the ribosomal RNA gene amplicon sequencing to explore the diversity and flexibility of *Symbiodinium*. The result demonstrates that *G. fascicularis* at Hainan Island exhibits a high level of symbiont flexibility, and the changes in relative abundance of thermally tolerant *Symbiodinium* clade D associated with *G. fascicularis* are possibly driven by temperature. This finding implies that symbiont shuffling is likely a defensive mechanism of coral for local acclimatization to environmental changes.

## Materials and Methods

### Sample Collection

**FIGURE 1:**
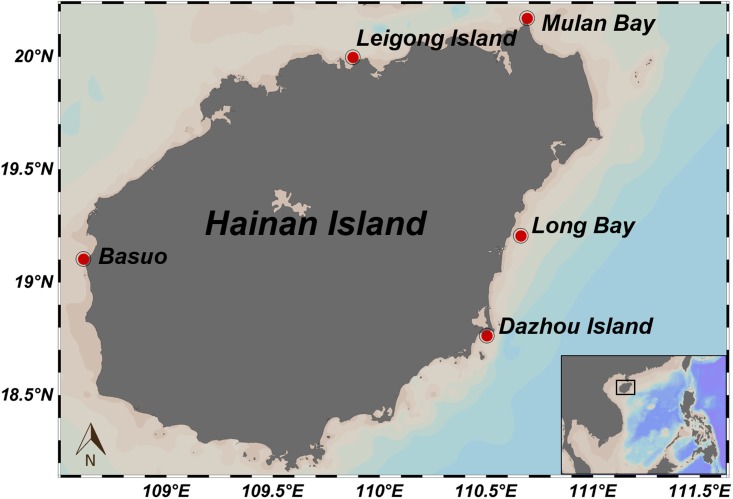
A geographic map showing five sampling locations at Hainan Island, China. Red dots represent sampling sites.

Samples of the scleractinian coral *G. fascicularis* were collected at depths between 2 and 4 m from the coast of Hainan Island in the South China Sea in September 2010 (**Figure 1**). At each location, six to seven colonies separated by at least 5 m were collected and fragments of approximately 4 cm^2^ were picked and preserved in 95% ethyl alcohol at field temperature and stored at -20°C until DNA extraction took place.

### DNA Extraction and Amplicon Sequencing

Total DNA was extracted as described previously (Zhou et al., 2012). The quality and quantity of the DNA were determined with a NanoDrop spectrophotometer (Thermo Fisher Scientific, United States). Purified DNA samples were stored at -20°C for future use.

All samples were PCR amplified using a pair of barcoded *Symbiodinium*-specific primers: ITSintfor2 (5^′^-GAATTGCAGAACTCCGTG-3^′^) and ITS2-reverse (5^′^-GGGATCCATATGCTTAAGTTCAGCGGGT-3^′^) (Lajeunesse and Trench, 2000) targeting the ITS2 region of the ribosomal RNA gene for *Symbiodinum.* PCR amplification was carried out on a thermocycle controller (Bio-Rad, United States) with the following program: initial denaturing at 94°C for 5 min; 35 cycles at 94°C for 30 s, 51°C for 30 s, and 72°C for 30 s; and a final extension at 72°C for 5 min. All PCR products were purified using the Qiagen Agarose Gel DNA Purification Kit (Qiangen, China) and quantified with the NanoDrop spectrophotometer. All amplification products were mixed in equal amount followed by sequencing on an Illumina Miseq platform using the 2 × 300 bp mode at Novogene (Beijing, China). The raw data were submitted to the NCBI Sequence Read Archive under accession number SRP066283.

### ITS2 Sequencing Data Processing

Overlapping paired-end reads were merged to obtain fragments using PEAR (Zhang et al., 2014). After de-multiplexing and quality control, a custom BLAST *Symbiodinium*-specific database of ITS2 types was downloaded (Arif et al., 2014), containing 408 ITS2 sequences. For each sample, datasets were randomly subsampled to 10,411 sequences (the lowest read number) which were subsequently searched against the database using BLASTn. Sequences were assigned to the ITS2 types that gave the highest identity in the BLASTn hits (Tong et al., 2017). The resulting counts of *Symbiodinium* ITS2 types were merged for downstream statistical analysis.

### Environmental Data

Aqua-MODIS sea surface temperature (SST) and chlorophyll *a* concentration (Chl *a*) with a spatial resolution of 4 km at each sampling location from January 2006 to December 2010 were obtained from NASA^1^.

### Statistical Analyses

The Shannon–Wiener (H^′^) diversity index was calculated to assess the level of alpha-diversity across samples from different locations. One-way analysis of variance and *post hoc* Tukey’s HDS comparisons were conducted to test the significance of differences in diversity between sampling locations. The similarity of *Symbiodinium* assemblages was also characterized by non-metric multidimensional scaling (nMDS) using the Bray–Curtis distance metric after data transformation. Analysis of variance (ADONIS) was performed to test the significance of differences in *Symbiodinium* communities among different sampling locations. The significant relationship between environmental variables (SST and Chl *a*) and *Symbiodinium* community composition was assessed using Monte Carlo permutation methods. All statistical analysis were conducted using the vegan package (Oksanen et al., 2015) in the R software environment (R 3.1.2).

## Results And Discussion

### *Symbiodinium* Community Diversity and Flexibility

**FIGURE 2:**
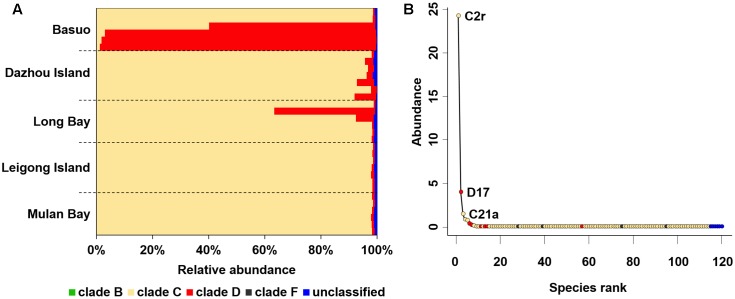
*Symbiodinium* compositions for complete dataset. **(A)** *Symbiodinium* compositions at clade level. Bars represent the percentage of each clade. **(B)** Distribution of taxonomic abundances among *Symbiodinium* subclades.

In total, 997,760 qualified sequences were obtained from 32 samples (10,411–51,626 sequences per sample). A total of 119 *Symbiodinium* ITS2 subclades were assigned based on alignment with the ITS2 database at the 97% similarity level, covering clades B, C, D, and F. Overall, clade C comprised the highest proportion of sequences (averagely 85.6%), followed by clade D (averagely 13.6%) and then rare clades B and F (**Figure 2A**). C2r and D17 were the most dominant ITS2 subclades representing > 99% of the sequences for all samples. All individual colonies contained multiple *Symbiodinium* subclades belonging to different clades. Despite a high number of distinct *Symbiodinium* types, most of them had abundances lower than 0.1% (**Figure 2B**), indicating that rare subclades are present in heterogeneous *Symbiodinium* assemblages.

It is believed that low abundances of cryptic *Symbiodinium* have largely been overlooked by conventional screening techniques (Mieog et al., 2009). An increasing body of evidence shows that highly diverse rare taxa with important ecological roles are prevalent elsewhere (Lynch and Neufeld, 2015) and are being increasingly explored in reef-building corals (Silverstein et al., 2012; Green et al., 2014; Quigley et al., 2014; Kennedy et al., 2015; Boulotte et al., 2016). The *Symbiodinium* types in clades B and F associated with *G. fascicularis* are unusual, which have rarely been reported from the South China Sea (Huang et al., 2011; Zhou and Huang, 2011; Zhou et al., 2012; Tong et al., 2017) or other regions (Lajeunesse et al., 2004, 2010a). The results presented here suggest that *G. fascicularis* exhibits a high cryptic diversity and flexibility in symbiotic associations. It has been shown that rare *Symbiodinium* types (e.g., type D1) have the potential to enable the coral host to resist heat stress through symbiont shuffling or switching (Lajeunesse et al., 2009; Silverstein et al., 2012, 2015; Bay et al., 2016; Boulotte et al., 2016). For example, *Acropora* can change its thermally tolerant symbiont abundance from rare to dominant in a response to heat stress (Berkelmans and Van Oppen, 2006). Community diversity and functional redundancy may contribute to the stability of community resistance and resilience (Oliver et al., 2015), which has been characterized in coral holobionts (Silverstein et al., 2012, 2015). Highly diverse and flexible *Symbiodinium* may facilitate the ability of *G. fascicularis* to survive successfully in various habitats they experience throughout the Indo-Pacific area. However, the real contribution of rare symbionts to the host coral and their ecological significance is still unclear and needs to be addressed in future (Lee et al., 2016).

The *G. fascicularis* holobiont can be viewed as a highly complex symbiotic system with the flexibility to associate with a wide range of *Symbiodinium* (Blackall et al., 2015). It has been suggested that the mode of transmission of symbionts can affect the flexibility of coral-algal symbiosis (Baker, 2003; Fabina et al., 2012). Therefore, horizontal transmission of endosymbionts in each generation may provide greater opportunities for *G. fascicularis* to obtain multiple symbionts from the external environment. However, emerging evidence shows that many corals can host multiple *Symbiodinium* subclades without correlation with the mode of transmission (van Oppen, 2004). In addition, other factors such as environmental variability, host recognition and maintenance mechanisms can also influence the flexibility of coral-algal associations (Baker, 2003; Rodriguez-Lanetty et al., 2006; Dunn and Weis, 2009). Moreover, the development of coral especially in early life stages has additional effects on symbiont acquisition and selection (Abrego et al., 2009; McIlroy and Coffroth, 2017; Zhou et al., 2017). For example, Abrego et al. (2009) demonstrated that the symbiont associations in juvenile *Acropora* are more flexible than those in adults.

### Temperature Drives the *Symbiodinium* Assemblages in *G. fascicularis*

**FIGURE 3:**
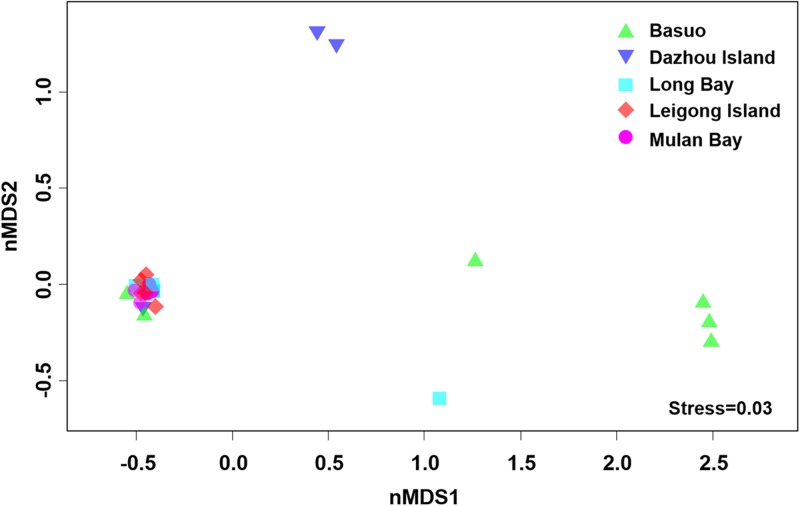
Non-metric multidimensional scaling (nMDS) plotting of *Symbiodinium* communities using subclade data among locations. Axes do not represent any measured parameters, but define a 2-D space that allow the best spatial representation of sample similarity, based on Bray–Curtis similarity indices.

**FIGURE 4:**
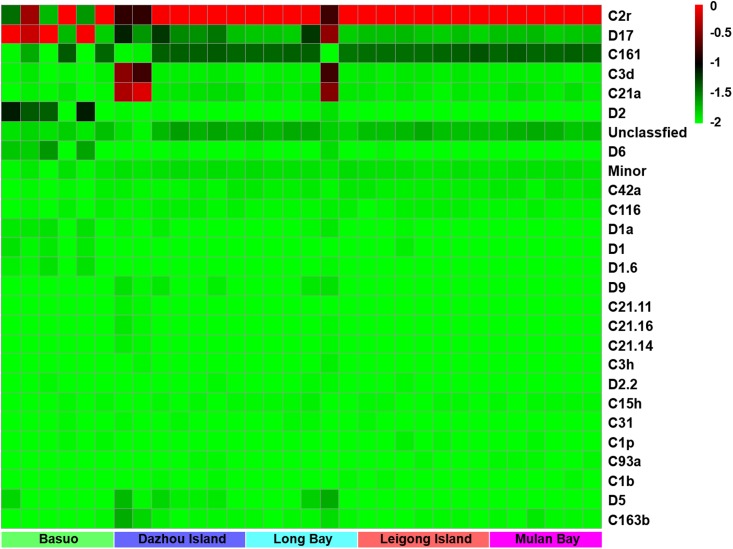
A heatmap visualization of the dominant subclades of *Symbiodinium* (relative abundance > 0.1% in one sample at least). The top scales “–2, –1.5, –1, –0.5, and 0” showed the relative abundance of “0, 2, 9, and 99%,” respectively.

No significant differences in the Shannon diversity index were detected among sampling locations (one-way ANOVA; *F* = 1.618, *p* = 0.198). However, there were significant differences in the *Symbiodinium* assemblages between sampling locations (**Figure 3**; ADONIS, *p* = 0.01), demonstrating that coral-algal symbiosis is highly flexible around Hainan Island. Importantly, these differences were attributed to changes in the relative abundance of existing *Symbiodinium* types in individuals. Of six colonies of *G. fascicularis* at Basuo, four were dominated by clade D with sparse clade C, whereas the others contained abundant clade C with rare clade D. At subclade level, D17 dominated at Basuo, whereas C2r dominated in other locations (**Figure 4**). The abundance of each of the D1a, D2, and D6 subclades was higher at Basuo than at other locations, indicating that these holobionts are likely to be locally adapted through shifts in symbiont community composition.

**FIGURE 5:**
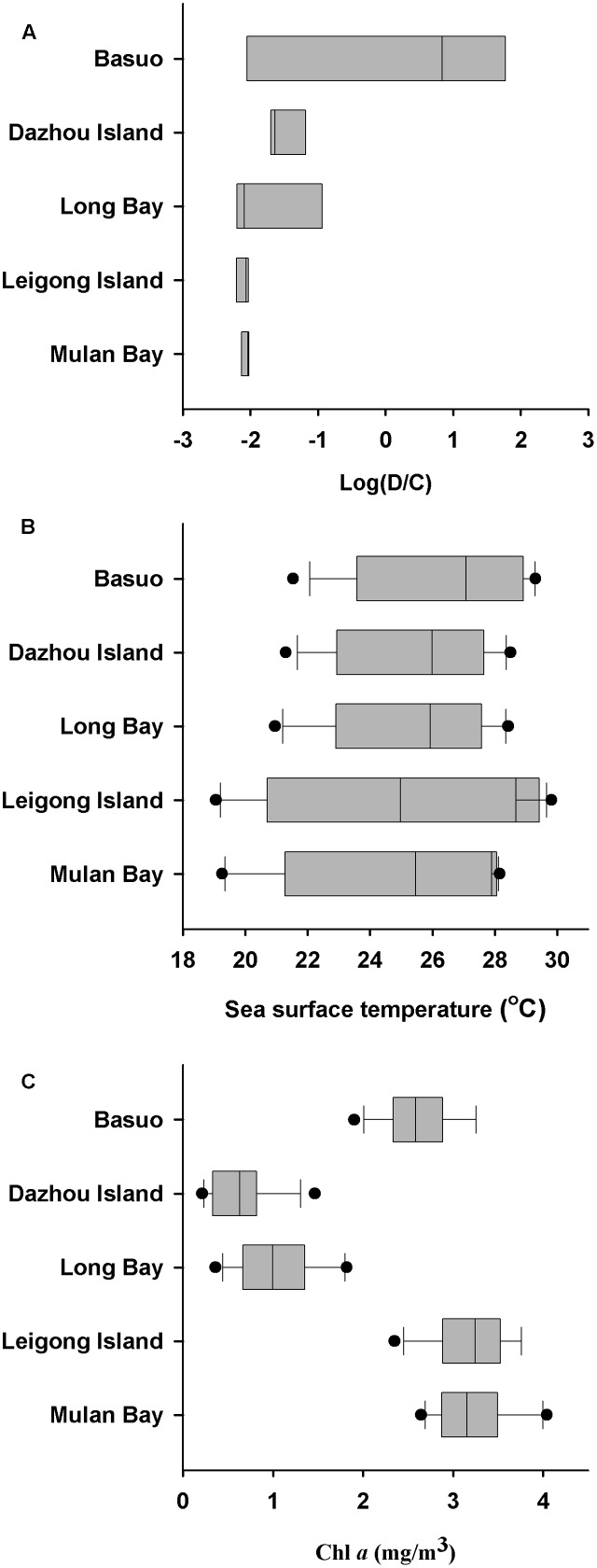
Changes in dominant *Symbiodinium* clades, sea surface temperature (SST), and chlorophyll *a* (Chl *a*) concentrations. **(A)** Log ratio of *Symbiodinium* clade D/C relative abundance at each location. **(B)** Variation of SST at each location. **(C)** Variation of Chl *a* concentrations at each location. Average monthly satellite measurements (SST and Chl *a*) from January 2006 to December 2010 were acquired from the Giovanni online data system, which is maintained by the NASA Goddard Earth Sciences Data and Information Services Center. Each dot represents the monthly average ± SD. Boxplots showing median, first and third quartiles, and maximum and minimum seasonal ranges by location.

Sea surface temperatures and Chl *a* concentrations from 2006 to 2010 at each location were employed to investigate the relationships between environmental conditions and *Symbiodinium* communities (**Figure 5**). Monthly average SSTs at Mulan Bay, Long Bay and Dazhou Island decreased sharply in July, possibly due to the Qiongdong Upwelling in summer (Jing et al., 2015). Of the sample locations, Leigong Island experienced the largest annual fluctuation in monthly average SST (∼11°C). Annual average SSTs was highest at Basuo (26.4°C), followed by Dazhou Island (25.5°C), Long Bay (25.3°C), Leigong Island (24.7°C), and Mulan Bay (24.5°C). The monthly average Chl *a* concentrations of all the locations showed little variation throughout the year, but Long Bay and Dazhou Island had lower yearly average Chl *a* concentrations. *Symbiodinium* communities at Basuo was significantly correlated with spring average SSTs (Monte Carlo permutation test; *p* < 0.05), but there were no significant differences between *Symbiodinium* communities and Chl *a* concentrations (Monte Carlo permutation test; *p* > 0.05). *G. fascicularis* had a high specificity for *Symbiodinium* clade D at Basuo where annual average SSTs were the highest (**Figure 5**). In contrast, Chl *a* values at all locations did not show any patterns or trends consistent with observed *Symbiodinium* distribution patterns.

It is well-known that *Symbiodinium* clade D occurs more frequently in areas with high SST and high turbidity (e.g., Lajeunesse et al., 2010a; Keshavmurthy et al., 2012). Furthermore, it is evident that heat tolerant *Symbiodinium* can confer thermal tolerance to its host coral but at a cost of reduced growth rate, a decline in reproduction and increased susceptibility to disease (e.g., Little et al., 2004; Berkelmans and Van Oppen, 2006; Silverstein et al., 2015). It has been suggested that symbiont compositions may be regulated to maintain optimal benefit to the host in a given environment (Cunning et al., 2015a,b). Such trade-off mechanisms may depend on both biotic (e.g., host species, host ontogeny, and symbiont competition) and abiotic (e.g., temperature, light, and nutrients) factors, and may also involve stochastic processes and selective pressures. In the present study, relative high abundances of *Symbiodinium* clade D at Basuo where the annual average SST is the highest are possibly mediated by a cost-benefit trade-off by the coral *G. fascicularis*. These observations agree with our previous investigations (Huang et al., 2011; Tong et al., 2017), which reported that clade D in *G. fascicularis* was more prevalent in tropical locations than in subtropical locations from the South China Sea, which might be attributed to the latitudinal temperature gradients. It is known that temperature has a profound influence on the ecological structure of coral communities (Brown et al., 2004). Previous studies also found that temperature is the main determinant to the geographic distribution of *Symbiodinium* in both conspecific corals (Lajeunesse et al., 2010a) and local adaptation (Howells et al., 2012). We suggest that temperature is the main environmental factor influencing the spatial variability of *Symbiodinium* assemblages in *G. fascicularis* around Hainan Island. However, other undetermined environmental factors, such as light intensity, and nutrient levels may also contribute to the biogeographical patterns of host-*Symbiodinium* associations (Baker, 2003), which can be investigated thoroughly in the future.

A better understanding of the spatial patterns will allow us to predict how corals will respond to environmental change over time (Dunne et al., 2004). In the present study, it may also reflect the capacity of the coral *G. fascicularis* to respond to environmental disturbances (e.g., thermal bleaching) by shuffling its internal symbionts. Some coral-algal associations remain remarkably stable over time (Thornhill et al., 2009; Williams et al., 2015) or revert to their original status after thermal bleaching (Lajeunesse et al., 2010b), which can be explained by the trade-off mechanism (Cunning et al., 2015b). More recently, it has been shown that symbiont shuffling in reef-building corals is attributed to the magnitude of the disturbance and the recovery conditions (Cunning et al., 2015a; Silverstein et al., 2015). The combinations of long-term, *in situ* field observations and elaborate laboratory experiments may provide more supporting evidence for symbiont shuffling in a better understanding of how coral will adapt to future climate changes. With the advances in ‘omic’ technologies, it is becoming feasible to elucidate the molecular mechanism of local acclimation and symbiont shuffling by analyzing biochemical complementarity of the symbiotic partners (e.g., Lin et al., 2015).

## Conclusion

This study characterized the geographic patterns of host-*Symbiodinium* associations in an ecologically important scleractinian coral *G. fascicularis* using high-throughput sequencing of ITS2 amplicons. We confirmed that *G. fascicularis* at Hainan Island exhibits a high level of symbiont flexibility, with the thermally tolerant *Symbiodinium* types in clade D being prevalent and highly abundant at locations with the highest annual average SSTs. These findings suggest that symbiont shuffling has the potential to serve as a trade-off mechanism for local acclimatization in *G. fascicularis*. The present study provides a better understanding of *Symbiodinium* diversity and distribution, which is important to predict the persistence of coral-algal associations in the presence of increasing environmental perturbations such as global warming.

## Author Contributions

GZ, SL, P-YQ, and HH designed the study. YL collected samples. HT, LJ, YZ, XL, and MG performed experiments. GZ and LC analyzed data. GZ wrote the paper. All authors revised and approved the manuscript.

## Conflict of Interest Statement

The authors declare that the research was conducted in the absence of any commercial or financial relationships that could be construed as a potential conflict of interest.

## Footnotes

1 https://modis.gsfc.nasa.gov/

## References

[B1] Abrego D. Van Oppen M. J. H. Willis B. L. (2009). Highly infectious symbiont dominates initial uptake in coral juveniles. *Mol. Ecol.* 18 3518–3531. 10.1111/j.1365-294X.2009.04275.x 19627495

[B2] Arif C. Daniels C. Bayer T. Banguera-Hinestroza E. Barbrook A. Howe C. J. (2014). Assessing *Symbiodinium* diversity in scleractinian corals via next-generation sequencing-based genotyping of the ITS2 rDNA region. *Mol. Ecol.* 23 4418–4433. 10.1111/mec.12869 25052021 PMC4285332

[B3] Baird A. H. Cumbo V. R. Leggat W. Rodriguez-Lanetty M. (2007). Fidelity and flexibility in coral symbioses. *Mar. Ecol. Prog. Ser.* 347 307–309. 10.3354/meps07220

[B4] Baker A. C. (2003). Flexibility and specificity in coral-algal symbiosis: diversity, ecology, and biogeography of *Symbiodinium*. *Annu. Rev. Ecol. Evol. Syst.* 34 661–689. 10.1146/annurev.ecolsys.34.011802.132417

[B5] Baker A. C. Starger C. J. Mcclanahan T. R. Glynn P. W. (2004). Corals’ adaptive response to climate change. *Nature* 430 741. 10.1038/430741a 15306799

[B6] Bay L. K. Doyle J. Logan M. Berkelmans R. (2016). Recovery from bleaching is mediated by threshold densities of background thermo-tolerant symbiont types in a reef-building coral. *R. Soc. Open Sci.* 3:160322. 10.1098/rsos.160322 27429786 PMC4929921

[B7] Berkelmans R. Van Oppen M. J. H. (2006). The role of zooxanthellae in the thermal tolerance of corals: a ‘nugget of hope’ for coral reefs in an era of climate change. *Proc. Biol. Sci.* 273 2305–2312. 10.1098/rspb.2006.3567 16928632 PMC1636081

[B8] Blackall L. L. Wilson B. Van Oppen A. J. H. (2015). Coral-the world’s most diverse symbiotic ecosystem. *Mol. Ecol.* 24 5330–5347. 10.1111/mec.13400 26414414

[B9] Boulotte N. M. Dalton S. J. Carroll A. G. Harrison P. L. Putnam H. M. Peplow L. M. (2016). Exploring the *Symbiodinium* rare biosphere provides evidence for symbiont switching in reef-building corals. *ISME J.* 10 2693–2701. 10.1038/ismej.2016.54 27093048 PMC5113844

[B10] Brading P. Warner M. E. Davey P. Smith D. J. Achterberg E. P. Suggett D. J. (2011). Differential effects of ocean acidification on growth and photosynthesis among phylotypes of *Symbiodinium* (Dinophyceae). *Limnol. Oceanogr.* 56 927–938. 10.4319/lo.2011.56.3.0927

[B11] Brown J. H. Gillooly J. F. Allen A. P. Savage V. M. West G. B. (2004). Toward a metabolic theory of ecology. *Ecology* 85 1771–1789. 10.1890/03-9000

[B12] Buddemeier R. W. Fautin D. G. (1993). Coral bleaching as an adaptive mechanism - a testable hypothesis. *Bioscience* 43 320–326.

[B13] Cunning R. Silverstein R. N. Baker A. C. (2015a). Investigating the causes and consequences of symbiont shuffling in a multi-partner reef coral symbiosis under environmental change. *Proc. Biol. Sci.* 282:20141725. 10.1098/rspb.2014.1725 26041354 PMC4590431

[B14] Cunning R. Vaughan N. Gillette P. Capo T. R. Mate J. L. Baker A. C. (2015b). Dynamic regulation of partner abundance mediates response of reef coral symbioses to environmental change. *Ecology* 96 1411–1420. 10.1890/14-0449.1 26236853

[B15] Cunning R. Yost D. M. Guarinello M. L. Putnam H. M. Gates R. D. (2015c). Variability of *Symbiodinium* communities in waters, sediments, and corals of thermally distinct reef pools in American Samoa. *PLOS ONE* 10:e0145099. 10.1371/journal.pone.0145099 26713847 PMC4695085

[B16] Dunn S. R. Weis V. M. (2009). Apoptosis as a post-phagocytic winnowing mechanism in a coral-dinoflagellate mutualism. *Environ. Microbiol.* 11 k268–276. 10.1111/j.1462-2920.2008.01774.x 19125818

[B17] Dunne J. A. Saleska S. R. Fischer M. L. Harte J. (2004). Integrating experimental and gradient methods in ecological climate change research. *Ecology* 85 904–916. 10.1890/03-8003

[B18] Fabina N. S. Putnam H. M. Franklin E. C. Stat M. Gates R. D. (2012). Transmission mode predicts specificity and interaction patterns in coral-*Symbiodinium* networks. *PLOS ONE* 7:e44970. 10.1371/journal.pone.0044970 23028711 PMC3445617

[B19] Green E. A. Davies S. Matz M. V. Medina M. (2014). Quantifying cryptic *Symbiodinium* diversity within *Orbicella faveolata* and *Orbicella franksi* at the Flower Garden Banks, Gulf of Mexico. *PeerJ* 2:e386. 10.7717/peerj.386 24883247 PMC4034615

[B20] Howells E. J. Beltran V. H. Larsen N. W. Bay L. K. Willis B. L. Van Oppen M. J. H. (2012). Coral thermal tolerance shaped by local adaptation of photosymbionts. *Nat. Clim. Change* 2 116–120. 10.1038/nclimate1330

[B21] Huang H. Dong Z. J. Huang L. M. Yang J. H. Di B. P. Li Y. C. (2011). Latitudinal variation in algal symbionts within the scleractinian coral *Galaxea fascicularis* in the South China Sea. *Mar. Biol. Res.* 7 208–211. 10.1080/17451000.2010.489616

[B22] Jing Z. Y. Qi Y. Q. Du Y. Zhang S. W. Xie L. L. (2015). Summer upwelling and thermal fronts in the northwestern South China Sea: observational analysis of two mesoscale mapping surveys. *J. Geophys. Res. Oceans* 120 1993–2006. 10.1002/2014JC010601

[B23] Jones A. M. Berkelmans R. Van Oppen M. J. H. Mieog J. C. Sinclair W. (2008). A community change in the algal endosymbionts of a scleractinian coral following a natural bleaching event: field evidence of acclimatization. *Proc. Biol. Sci.* 275 1359–1365. 10.1098/rspb.2008.0069 18348962 PMC2367621

[B24] Kennedy E. V. Foster N. L. Mumby P. J. Stevens J. R. (2015). Widespread prevalence of cryptic *Symbiodinium* D in the key Caribbean reef builder, *Orbicella annularis*. *Coral Reefs* 34 519–531. 10.1007/s00338-015-1264-4

[B25] Keshavmurthy S. Hsu C. M. Kuo C. Y. Meng P. J. Wang J. T. Chen C. L. A. (2012). Symbiont communities and host genetic structure of the brain coral *Platygyra verweyi*, at the outlet of a nuclear power plant and adjacent areas. *Mol. Ecol.* 21 4393–4407. 10.1111/j.1365-294X.2012.05704.x 22809041

[B26] Lajeunesse T. C. (2001). Investigating the biodiversity, ecology, and phylogeny of endosymbiotic dinoflagellates in the genus *Symbiodinium* using the ITS region: In search of a “species” level marker. *J. Phycol.* 37 866–880. 10.1046/j.1529-8817.2001.01031.x

[B27] Lajeunesse T. C. Bhagooli R. Hidaka M. Devantier L. Done T. Schmidt G. W. (2004). Closely related *Symbiodinium* spp. differ in relative dominance in coral reef host communities across environmental, latitudinal and biogeographic gradients. *Mar. Ecol. Prog. Ser.* 284 147–161. 10.3354/meps284147

[B28] Lajeunesse T. C. Pettay D. T. Sampayo E. M. Phongsuwan N. Brown B. Obura D. O. (2010a). Long-standing environmental conditions, geographic isolation and host-symbiont specificity influence the relative ecological dominance and genetic diversification of coral endosymbionts in the genus *Symbiodinium*. *J. Biogeogr.* 37 785–800. 10.1111/j.1365-2699.2010.02273.x

[B29] Lajeunesse T. C. Smith R. Walther M. Pinzon J. Pettay D. T. Mcginley M. (2010b). Host-symbiont recombination versus natural selection in the response of coral-dinoflagellate symbioses to environmental disturbance. *Proc. Biol. Sci.* 277 2925–2934. 10.1098/rspb.2010.0385 20444713 PMC2982020

[B30] Lajeunesse T. C. Smith R. T. Finney J. Oxenford H. (2009). Outbreak and persistence of opportunistic symbiotic dinoflagellates during the 2005 Caribbean mass coral ‘bleaching’ event. *Proc. Biol. Sci.* 276 4139–4148. 10.1098/rspb.2009.1405 19740874 PMC2821356

[B31] Lajeunesse T. C. Trench R. K. (2000). Biogeography of two species of *Symbiodinium* (Freudenthal) inhabiting the intertidal sea anemone *Anthopleura elegantissima* (Brandt). *Biol. Bull.* 199 126–134. 10.2307/1542872 11081711

[B32] Lee M. J. Jeong H. J. Jang S. H. Lee S. Y. Kang N. S. Lee K. H. (2016). Most low-abundance “background” *Symbiodinium* spp. are transitory and have minimal functional significance for symbiotic corals. *Microb. Ecol.* 71 771–783. 10.1007/s00248-015-0724-2 26781946

[B33] Lien Y. T. Keshavmurthy S. Nakano Y. Plathong S. Huang H. Hsu C. M. (2013). Host genetics and *Symbiodinium* D diversity in a stress-tolerant scleractinian coral, *Oulastrea crispata*, in the West Pacific. *Mar. Ecol. Prog. Ser.* 473 163–177. 10.3354/meps10041

[B34] Lin S. J. Cheng S. F. Song B. Zhong X. Lin X. Li W. J. (2015). The *Symbiodinium kawagutii* genome illuminates dinoflagellate gene expression and coral symbiosis. *Science* 350 691–694. 10.1126/science.aad0408 26542574

[B35] Little A. F. Van Oppen M. J. H. Willis B. L. (2004). Flexibility in algal endosymbioses shapes growth in reef corals. *Science* 304 1492–1494. 10.1126/science.1095733 15178799

[B36] Lynch M. D. Neufeld J. D. (2015). Ecology and exploration of the rare biosphere. *Nat. Rev. Microbiol.* 13 217–229. 10.1038/nrmicro3400 25730701

[B37] McIlroy S. E. Coffroth M. A. (2017). Coral ontogeny affects early symbiont acquisition in laboratory-reared recruits. *Coral Reefs* 36 927–932. 10.1007/s00338-017-1584-7

[B38] Mieog J. C. Van Oppen M. J. H. Berkelmans R. Stam W. T. Olsen J. L. (2009). Quantification of algal endosymbionts (*Symbiodinium*) in coral tissue using real-time PCR. *Mol. Ecol. Resour.* 9 74–82. 10.1111/j.1755-0998.2008.02222.x 21564569

[B39] Oksanen J. Blanchet F. G. Kindt R. Legendre P. Minchin P. R. O’Hara R. B. (2015). *Vegan: Community Ecology Package. R Package Version 2.3-0.* Available at: http://CRAN.R-project.org/package=vegan

[B40] Oliver T. H. Heard M. S. Isaac N. J. B. Roy D. B. Procter D. Eigenbrod F. (2015). Biodiversity and resilience of ecosystem functions. *Trends. Ecol. Evol.* 30 673–684. 10.1016/j.tree.2015.08.009 26437633

[B41] Pandolfi J. M. Connolly S. R. Marshall D. J. Cohen A. L. (2011). Projecting coral reef futures under global warming and ocean acidification. *Science* 333 418–422. 10.1126/science.1204794 21778392

[B42] Pochon X. Gates R. D. (2010). A new *Symbiodinium* clade (Dinophyceae) from soritid foraminifera in Hawai’i. *Mol. Phylogenet. Evol.* 56 492–497. 10.1016/j.ympev.2010.03.040 20371383

[B43] Putnam H. M. Stat M. Pochon X. Gates R. D. (2012). Endosymbiotic flexibility associates with environmental sensitivity in scleractinian corals. *Proc. Biol. Sci.* 279 4352–4361. 10.1098/rspb.2012.1454 22933373 PMC3479799

[B44] Quigley K. M. Davies S. W. Kenkel C. D. Willis B. L. Matz M. V. Bay L. K. (2014). Deep-sequencing method for quantifying background abundances of *Symbiodinium* types: exploring the rare *Symbiodinium* biosphere in reef-building corals. *PLOS ONE* 9:e94297. 10.1371/journal.pone.0094297 24728373 PMC3984134

[B45] Rodriguez-Lanetty M. Wood-Charlson E. M. Hollingsworth L. L. Krupp D. A. Weis V. M. (2006). Temporal and spatial infection dynamics indicate recognition events in the early hours of a dinoflagellate/coral symbiosis. *Mar. Biol.* 149 713–719. 10.1007/s00227-006-0272-x

[B46] Sampayo E. M. Franceschinis L. Hoegh-Guldberg O. Dove S. (2007). Niche partitioning of closely related symbiotic dinoflagellates. *Mol. Ecol.* 16 3721–3733. 10.1111/j.1365-294X.2007.03403.x 17845444

[B47] Silverstein R. N. Correa A. M. S. Baker A. C. (2012). Specificity is rarely absolute in coral-algal symbiosis: implications for coral response to climate change. *Proc. Biol. Sci.* 279 2609–2618. 10.1098/rspb.2012.0055 22367985 PMC3350700

[B48] Silverstein R. N. Cunning R. Baker A. C. (2015). Change in algal symbiont communities after bleaching, not prior heat exposure, increases heat tolerance of reef corals. *Glob. Change Biol.* 21 236–249. 10.1111/gcb.12706 25099991

[B49] Tchernov D. Gorbunov M. Y. De Vargas C. Yadav S. N. Milligan A. J. Haggblom M. (2004). Membrane lipids of symbiotic algae are diagnostic of sensitivity to thermal bleaching in corals. *Proc. Natl. Acad. Sci. U.S.A.* 101 13531–13535. 10.1073/pnas.0402907101 15340154 PMC518791

[B50] Thomas L. Kendrick G. A. Kennington W. J. Richards Z. T. Stat M. (2014). Exploring *Symbiodinium* diversity and host specificity in *Acropora* corals from geographical extremes of Western Australia with 454 amplicon pyrosequencing. *Mol. Ecol.* 23 3113–3126. 10.1111/mec.12801 24845644

[B51] Thornhill D. J. Xiang Y. Fitt W. K. Santos S. R. (2009). Reef endemism, host specificity and temporal stability in populations of symbiotic dinoflagellates from two ecologically dominant Caribbean corals. *PLOS ONE* 4:e6262. 10.1371/journal.pone.0006262 19603078 PMC2706050

[B52] Tong H. Y. Cai L. Zhou G. W. Yuan T. Zhang W. P. Tian R. M. (2017). Temperature shapes coral-algal symbiosis in the South China Sea. *Sci. Rep.* 7:40118. 10.1038/srep40118 28084322 PMC5234030

[B53] van Oppen M. J. H. (2004). Mode of zooxanthella transmission does not affect zooxanthella diversity in acroporid corals. *Mar. Biol.* 144 1–7. 10.1007/s00227-003-1187-4 10614872

[B54] Williams A. D. Brown B. E. Putchim L. Sweet M. J. (2015). Age-related shifts in bacterial diversity in a reef coral. *PLOS ONE* 10:0144902. 10.1371/journal.pone.0144902 26700869 PMC4689413

[B55] Zhang J. J. Kobert K. Flouri T. Stamatakis A. (2014). PEAR: a fast and accurate Illumina Paired-End reAd mergeR. *Bioinformatics* 30 614–620. 10.1093/bioinformatics/btt593 24142950 PMC3933873

[B56] Zhou G. W. Cai L. Yuan T. Tian R. M. Tong H. Y. Zhang W. P. (2017). Microbiome dynamics in early life stages of the scleractinian coral *Acropora gemmifera* in response to elevated *p*CO_2_. *Environ. Microbiol.* 19 3342–3352. 10.1111/1462-2920.13840 28631353

[B57] Zhou G. W. Huang H. (2011). Low genetic diversity of symbiotic dinoflagellates (*Symbiodinium*) in scleractinian corals from tropical reefs in southern Hainan Island, China. *J. Syst. Evol.* 49 598–605. 10.1111/j.1759-6831.2011.00161.x

[B58] Zhou G. W. Huang H. Lian J. S. Zhang C. L. Li X. B. (2012). Habitat correlation of *Symbiodinium* diversity in two reef-building coral species in an upwelling region, eastern Hainan Island, China. *J. Mar. Biol. Assoc. U.K.* 92 1309–1316. 10.1017/S0025315411001548

[B59] Ziegler M. Arif C. Burt J. A. Dobretsov S. Roder C. Lajeunesse T. C. (2017). Biogeography and molecular diversity of coral symbionts in the genus *Symbiodinium* around the Arabian Peninsula. *J. Biogeogr.* 44 674–686. 10.1111/jbi.12913 28286360 PMC5324606

